# Bis(tetra­phenyl­phospho­nium) tris­[*N*-(methyl­sulfon­yl)dithio­carbimato(2−)-κ^2^ *S*,*S*′]stannate(IV)

**DOI:** 10.1107/S1600536809034114

**Published:** 2009-09-05

**Authors:** João P. Barolli, Marcelo R. L. Oliveira, Rodrigo S. Corrêa, Javier Ellena

**Affiliations:** a Departamento de Química, UFV, 36570-000 Viçosa, MG, Brazil; b Instituto de Física de São Carlos, Universidade de São Paulo, 13560-970, São Carlos, SP, Brazil

## Abstract

In the title complex, (C_24_H_20_P)_2_[Sn(C_2_H_3_NO_2_S_3_)_3_], the Sn^IV^ atom is coordinated by three *N*-(methyl­sulfon­yl)dithio­carbimate bidentate ligands through the anionic S atoms in a slightly distorted octa­hedral coordination geometry. There is one half-mol­ecule in the asymmetric unit; the complex is located on a crystallographic twofold rotation axis passing through the cation and bis­ecting one of the (non-symmetric) ligands, which appears thus disordered over two sites of equal occupancy. In the crystal structure, weak inter­molecular C—H⋯O and C—H⋯S inter­actions contribute to the packing stabilization.

## Related literature

For general background to tin(IV) dithio­carbamates, see: Barone *et al.* (2002 ▶); Coucouvanis (1979 ▶); Heard (2005 ▶); Menezes *et al.* (2005 ▶); Seth *et al.* (1992 ▶). For related structures of transition metal (Ni, Pt and Zn) complexes with dithio­carbimates derived from sulfonamides, see: Alves *et al.* (2009 ▶); Amim *et al.* (2008 ▶); Franca *et al.* (2006 ▶); Menezes *et al.* (2005 ▶). For the ligand synthesis, see: Hartke (1966 ▶).
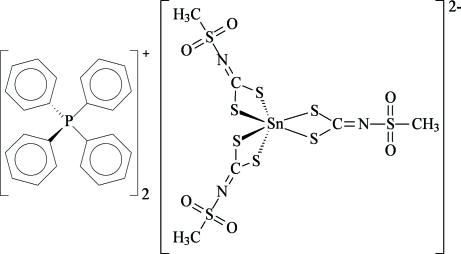


## Experimental

### 

#### Crystal data

(C_24_H_20_P)_2_[Sn(C_2_H_3_NO_2_S_3_)_3_]
*M* *_r_* = 1305.13
Monoclinic, 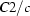

*a* = 18.5563 (3) Å
*b* = 13.6096 (2) Å
*c* = 23.3203 (3) Å
β = 91.355 (1)°
*V* = 5887.75 (15) Å^3^
*Z* = 4
Mo *K*α radiation
μ = 0.86 mm^−1^
*T* = 298 K
0.40 × 0.11 × 0.07 mm

#### Data collection

Nonius KappaCCD diffractometer
Absorption correction: gaussian (Coppens *et al.*, 1965 ▶) *T*_min_ = 0.726, *T*_max_ = 0.943
17695 measured reflections
5178 independent reflections
4871 reflections with *I* > 2σ(*I*)
*R*_int_ = 0.049

#### Refinement

*R*[*F* ^2^ > 2σ(*F* ^2^)] = 0.068
*wR*(*F* ^2^) = 0.138
*S* = 1.25
5178 reflections
372 parameters
1 restraint
H-atom parameters constrained
Δρ_max_ = 0.62 e Å^−3^
Δρ_min_ = −0.80 e Å^−3^

### 

Data collection: *COLLECT* (Nonius, 2000 ▶); cell refinement: *DENZO* and *SCALEPACK* (Otwinowski & Minor, 1997 ▶); data reduction: *DENZO* and *SCALEPACK*; program(s) used to solve structure: *SHELXS97* (Sheldrick, 2008 ▶); program(s) used to refine structure: *SHELXL97* (Sheldrick, 2008 ▶); molecular graphics: *ORTEP-3 for Windows* (Farrugia, 1997 ▶) and *Mercury* (Macrae *et al.*, 2006 ▶); software used to prepare material for publication: *WinGX* (Farrugia, 1999 ▶).

## Supplementary Material

Crystal structure: contains datablocks global, I. DOI: 10.1107/S1600536809034114/bg2277sup1.cif

Structure factors: contains datablocks I. DOI: 10.1107/S1600536809034114/bg2277Isup2.hkl

Additional supplementary materials:  crystallographic information; 3D view; checkCIF report

## Figures and Tables

**Table 1:** Hydrogen-bond geometry (Å, °)

*D*—H⋯*A*	*D*—H	H⋯*A*	*D*⋯*A*	*D*—H⋯*A*
C2—H2*B*⋯O4^i^	0.96	2.35	3.284 (13)	166
C16—H16⋯O3^ii^	0.93	2.60	3.2203 (10)	125
C19—H19⋯O1^iii^	0.93	2.47	3.296 (7)	148
C28—H28⋯S4^ii^	0.93	2.69	3.345 (5)	128

Symmetry codes: (i) 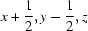; (ii) 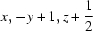; (iii) 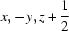.

**Fig. 1.**
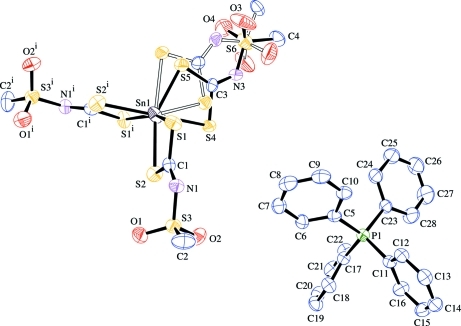
Structure of the complex showing atom labels, with ellipsoids drawn at the 30% probability level. One of the two moieties in the disordered ligand is presented in open bonds. For clarity, H atoms have been omitted. [Symmetry code: i= -x, y, 1/2-z].

**Fig. 2.**
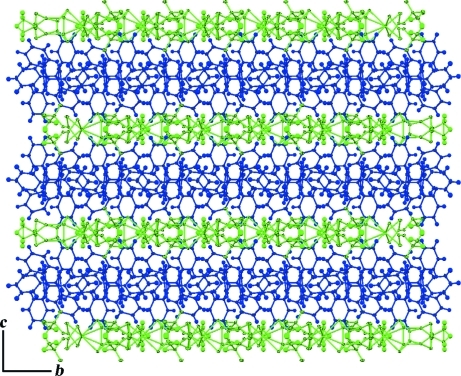
Crystal packing of the title compound forming two independents sheets. The complex are displayed in green and the phosphonium in blue.

## supplementary crystallographic information

### Comment

We became interested in the syntheses and characterization of tin(IV) dithiocarbimate complexes due to their similarity with the dithiocarbamate analogues, which have shown antifungal activity (Menezes *et al.*, 2005). Tin dithiocarbamates have also been used as molecular tin sulfide precursors for semiconductor films (Barone *et al.*, 2002). To the best of our knowledge, the title compound is the first member of a class of Sn complexes with general formula [Sn(RSO_2_N=CS_2_)_3_]^2-^. This class is related to tin(IV) dithiocarbamates (Coucouvanis, 1979; Heard, 2005 and Seth *et al.*, 1992). However, differently from the dithiocarbamates, these are anionic species. Some crystallographic structures of transition metal (Ni, Pt and Zn) complexes with dithiocarbimates derivated from sulfonamides are described in the literature (Alves *et al.*,2009; Amim *et al.*, 2008 and Franca *et al.*; 2006).

The title compound, which is quite stable under ambient conditions, comprises a complex dianion and two tetraphenylphosphonium cations, with the formula (Ph_4_P)_2_[Sn(CH_3_SO_2_N=CS_2_)_2_] (scheme). To the best of our knowledge the tris(methyldithiocarbimato)estannate(IV) anion is the first example of tin complexes with dithiocarbimate ligands derived from sulfonamides. So, in this paper we report the crystal structure of the title compound. The complex presents an octahedral environment around the Sn^IV^ atom with the ligands coordinating in a relatively distorted manner (Figure 1). The Sn—S bond lengths lie within the range 2.443 (3)–2.646 (2) Å. In the chelate rings the C—S fragments present bond lengths which are characteristic of a single bond [1.75 (1)–1.77 (1) Å]. These values are in agreement with related structures (Menezes *et al.*, 2005). One of the ligands appears disordered into two sites (arounf the twofold symmetry axis) with occupancy factor 0.5. Weak intermolecular C—H···O and C—H···S interactions contribute to packing stabilization (Table 1). Figure 2 shows a crystal packing view of the complex projected onto the *bc* plane, where two independent sheets are clearly visible: one of them formed by the complex (green in Figure 2) and another defined by phosphonium units (blue in Figure 2). Both sheets are linked by weak hydrogen bonds (Table 1).

### Experimental

The potassium methylsulfonyldithiocarbimate dihydrate was prepared from methanesulfonamide as described in the literature (Hartke,1966). The compound (1) was prepared in DMF (10 ml). Tin(IV) iodide (0.7 mmol) was added to a suspension of the potassium methylsulfonyldithiocarbimate dihydrate (2.1 mmol). The mixture was stirred for 1.5 h at room temperature and filtered. Water (15 ml) and tetraphenylphosphonium bromide (1.4 mmol) were added to the solution obtained. The mixture was stirred for 15 min and the solid product obtained was filtered, washed with distilled water and dried under reduced pressure for 1 day, yielding (Ph_4_P)_2_[Sn(CH_3_SO_2_N=CS_2_)_3_] (*ca *70%). Suitable crystals of (1) were obtained by slow evaporation of the solution of the compound in methanol/water (1:1 *v*/*v*); m. pt 420.6–422.0 K. Analysis found: C49.69, H 3.91, N 3.04%; C_54_H_49_N_3_O_6_P_2_S_9_Sn requires: C 49.69, H 3.78, N 3.22%. IR (most important bands, cm^-1^): 1437 *v*(C=N); 1291 *v*_ass_(SO_2_); 1127 *v*_sim_(SO_2_); 938 *v*_ass_(CS_2_) and 317 *v*(SnS).

### Refinement

Refinement in Cc proved that the disorder around the two fold axis was not an artifact, thus confirming the correct space group as C2/c. Similarity restraints were applied to the disordered ligand in order to to ensure a reasonable geometry. H atoms were positioned geometrically and refined as riding. C_aryl_—H = 0.93 Å, C_methyl_—H= 0.96 Å. *U*_iso_(H)= 1.2*U*_eq_(C_aryl_) or *U*_iso_(H) = 1.5*U*_eq_(C_methyl_).

### Crystal data

**Table:**

(C_24_H_20_P)_2_[Sn(C_2_H_3_NO_2_S_3_)_3_]	*F*(000) = 2664
*M**_r_* = 1305.13	*D*_x_ = 1.472 Mg m^−^^3^
Monoclinic, *C*2/*c*	Mo *K*α radiation, λ = 0.71073 Å
Hall symbol: -C 2yc	Cell parameters from 37024 reflections
*a* = 18.5563 (3) Å	θ = 2.9–26.4°
*b* = 13.6096 (2) Å	µ = 0.86 mm^−^^1^
*c* = 23.3203 (3) Å	*T* = 298 K
β = 91.355 (1)°	Prism, colourless
*V* = 5887.75 (15) Å^3^	0.40 × 0.11 × 0.07 mm
*Z* = 4	

### Data collection

**Table:**

Nonius KappaCCD diffractometer	4871 reflections with *I* > 2σ(*I*)
CCD rotation images, thick slices scans	*R*_int_ = 0.049
Absorption correction: gaussian (Coppens *et al.*, 1965)	θ_max_ = 25.0°, θ_min_ = 3.2°
*T*_min_ = 0.726, *T*_max_ = 0.943	*h* = −22→22
17695 measured reflections	*k* = −16→15
5178 independent reflections	*l* = −27→27

### Refinement

**Table:**

Refinement on *F*^2^	1 restraint
Least-squares matrix: full	H-atom parameters constrained
*R*[*F*^2^ > 2σ(*F*^2^)] = 0.068	*w* = 1/[σ^2^(*F*_o_^2^) + (0.0524*P*)^2^ + 7.2513*P*] where *P* = (*F*_o_^2^ + 2*F*_c_^2^)/3
*wR*(*F*^2^) = 0.138	(Δ/σ)_max_ < 0.001
*S* = 1.25	Δρ_max_ = 0.62 e Å^−^^3^
5178 reflections	Δρ_min_ = −0.80 e Å^−^^3^
372 parameters	

### Special details

**Table:**

Geometry. All e.s.d.'s (except the e.s.d. in the dihedral angle between two l.s. planes) are estimated using the full covariance matrix. The cell e.s.d.'s are taken into account individually in the estimation of e.s.d.'s in distances, angles and torsion angles; correlations between e.s.d.'s in cell parameters are only used when they are defined by crystal symmetry. An approximate (isotropic) treatment of cell e.s.d.'s is used for estimating e.s.d.'s involving l.s. planes.

### Fractional atomic coordinates and isotropic or equivalent isotropic displacement parameters (Å^2^)

**Table:**

	*x*	*y*	*z*	*U*_iso_*/*U*_eq_	Occ. (<1)
C1	0.1377 (2)	0.3823 (4)	0.23827 (19)	0.0512 (11)	
C2	0.3047 (4)	0.2250 (6)	0.2112 (3)	0.097 (2)	
H2A	0.2987	0.1898	0.2464	0.146*	
H2B	0.3355	0.2807	0.218	0.146*	
H2C	0.3261	0.1825	0.1835	0.146*	
C3	0.0123 (6)	0.7091 (10)	0.2265 (5)	0.054 (3)	0.5
C4	0.0208 (8)	0.9908 (8)	0.2192 (6)	0.084 (4)	0.5
H4A	0	1.0435	0.25	0.126*	
H4B	0.0692	0.9963	0.2088	0.126*	0.5
H4C	−0.01	0.9968	0.1853	0.126*	0.5
C5	0.1135 (3)	0.1693 (4)	0.42704 (19)	0.0513 (11)	
C6	0.1645 (3)	0.0950 (4)	0.4278 (2)	0.0630 (13)	
H6	0.1825	0.071	0.4626	0.076*	
C7	0.1888 (4)	0.0563 (4)	0.3767 (3)	0.0807 (17)	
H7	0.2227	0.006	0.3773	0.097*	
C8	0.1626 (4)	0.0925 (5)	0.3254 (3)	0.0826 (19)	
H8	0.1788	0.0661	0.2912	0.099*	
C9	0.1128 (3)	0.1669 (5)	0.3238 (2)	0.0737 (16)	
H9	0.0957	0.1912	0.2888	0.088*	
C10	0.0883 (3)	0.2056 (4)	0.3743 (2)	0.0602 (13)	
H10	0.0546	0.2563	0.3733	0.072*	
C11	−0.0120 (2)	0.1986 (3)	0.50305 (17)	0.0482 (11)	
C12	−0.0622 (3)	0.1985 (4)	0.45823 (19)	0.0546 (12)	
H12	−0.0472	0.207	0.4208	0.065*	
C13	−0.1347 (3)	0.1858 (4)	0.4689 (2)	0.0617 (13)	
H13	−0.1682	0.1852	0.4386	0.074*	
C14	−0.1570 (3)	0.1742 (4)	0.5237 (2)	0.0656 (14)	
H14	−0.2057	0.1654	0.5307	0.079*	
C15	−0.1080 (3)	0.1753 (4)	0.5685 (2)	0.0715 (15)	
H15	−0.1237	0.1677	0.6058	0.086*	
C16	−0.0362 (3)	0.1877 (4)	0.5588 (2)	0.0654 (14)	
H16	−0.0034	0.1887	0.5896	0.079*	
C17	0.1296 (2)	0.1673 (4)	0.55250 (18)	0.0492 (11)	
C18	0.1161 (3)	0.0707 (4)	0.5669 (2)	0.0622 (13)	
H18	0.0847	0.0335	0.5441	0.075*	
C19	0.1486 (3)	0.0285 (4)	0.6148 (2)	0.0689 (14)	
H19	0.1392	−0.0366	0.6243	0.083*	
C20	0.1953 (3)	0.0845 (5)	0.6485 (2)	0.0722 (16)	
H20	0.2174	0.0568	0.6809	0.087*	
C21	0.2091 (3)	0.1798 (5)	0.6346 (2)	0.0731 (17)	
H21	0.2405	0.2167	0.6577	0.088*	
C22	0.1768 (3)	0.2223 (4)	0.5866 (2)	0.0622 (13)	
H22	0.1868	0.2873	0.5772	0.075*	
C23	0.0993 (3)	0.3510 (4)	0.4934 (2)	0.0541 (12)	
C24	0.1468 (3)	0.3929 (4)	0.4560 (3)	0.0711 (15)	
H24	0.1673	0.355	0.4275	0.085*	
C25	0.1640 (4)	0.4919 (4)	0.4611 (3)	0.092 (2)	
H25	0.1957	0.5206	0.4356	0.11*	
C26	0.1348 (4)	0.5465 (5)	0.5030 (4)	0.102 (2)	
H26	0.1471	0.6125	0.5067	0.122*	
C27	0.0883 (5)	0.5062 (5)	0.5395 (4)	0.114 (3)	
H27	0.0688	0.5445	0.5683	0.137*	
C28	0.0690 (4)	0.4077 (5)	0.5346 (3)	0.093 (2)	
H28	0.0356	0.3808	0.5592	0.111*	
N1	0.1997 (2)	0.3395 (3)	0.23680 (16)	0.0549 (10)	
N3	0.0164 (4)	0.8014 (7)	0.2123 (3)	0.059 (2)	0.5
O1	0.1729 (2)	0.1825 (3)	0.18192 (17)	0.0874 (13)	
O2	0.2311 (2)	0.3177 (3)	0.13255 (15)	0.0814 (12)	
O3	−0.0270 (8)	0.8837 (7)	0.1904 (4)	0.134 (5)	0.5
O4	−0.0792 (5)	0.8933 (8)	0.2542 (6)	0.132 (4)	0.5
P1	0.08248 (6)	0.22110 (9)	0.49273 (5)	0.0472 (3)	
S1	0.12124 (7)	0.45967 (11)	0.29633 (6)	0.0639 (4)	
S2	0.06587 (7)	0.37025 (12)	0.18873 (5)	0.0676 (4)	
S3	0.22079 (7)	0.26505 (11)	0.18510 (5)	0.0629 (4)	
S5	0.01886 (19)	0.6562 (3)	0.21037 (15)	0.0599 (8)	0.5
S4	0.04165 (16)	0.6248 (2)	0.17458 (13)	0.0629 (7)	0.5
S6	0	0.88818 (17)	0.25	0.0880 (7)	
Sn1	0	0.49240 (4)	0.25	0.0642 (2)	

### Atomic displacement parameters (Å^2^)

**Table:**

	*U*^11^	*U*^22^	*U*^33^	*U*^12^	*U*^13^	*U*^23^
C1	0.053 (3)	0.053 (3)	0.047 (2)	−0.009 (2)	−0.010 (2)	0.005 (2)
C2	0.091 (4)	0.108 (5)	0.092 (5)	0.040 (4)	−0.011 (4)	−0.022 (4)
C3	0.041 (6)	0.061 (9)	0.060 (7)	−0.007 (6)	0.001 (5)	0.002 (6)
C4	0.109 (10)	0.043 (7)	0.099 (9)	0.013 (6)	0.004 (7)	0.007 (6)
C5	0.059 (3)	0.047 (3)	0.047 (3)	−0.009 (2)	0.002 (2)	−0.002 (2)
C6	0.075 (3)	0.056 (3)	0.059 (3)	0.008 (3)	0.009 (2)	−0.001 (2)
C7	0.103 (5)	0.056 (4)	0.085 (4)	0.004 (3)	0.028 (4)	−0.012 (3)
C8	0.115 (5)	0.071 (4)	0.062 (4)	−0.029 (4)	0.026 (3)	−0.023 (3)
C9	0.095 (4)	0.076 (4)	0.050 (3)	−0.026 (4)	0.002 (3)	−0.003 (3)
C10	0.071 (3)	0.061 (3)	0.048 (3)	−0.009 (3)	0.002 (2)	−0.001 (2)
C11	0.056 (3)	0.047 (3)	0.041 (2)	−0.003 (2)	−0.003 (2)	−0.0003 (19)
C12	0.064 (3)	0.057 (3)	0.042 (2)	0.001 (2)	−0.008 (2)	0.000 (2)
C13	0.057 (3)	0.063 (3)	0.064 (3)	0.001 (2)	−0.017 (2)	−0.001 (3)
C14	0.051 (3)	0.066 (4)	0.080 (4)	−0.004 (3)	0.001 (3)	0.007 (3)
C15	0.064 (3)	0.090 (4)	0.061 (3)	−0.003 (3)	0.006 (3)	0.012 (3)
C16	0.057 (3)	0.089 (4)	0.049 (3)	−0.005 (3)	−0.008 (2)	0.006 (3)
C17	0.047 (2)	0.059 (3)	0.041 (2)	0.000 (2)	−0.0049 (19)	−0.004 (2)
C18	0.070 (3)	0.060 (4)	0.055 (3)	0.002 (3)	−0.015 (2)	−0.003 (2)
C19	0.075 (4)	0.070 (4)	0.061 (3)	0.013 (3)	−0.009 (3)	0.009 (3)
C20	0.058 (3)	0.106 (5)	0.051 (3)	0.019 (3)	−0.010 (2)	0.005 (3)
C21	0.055 (3)	0.115 (6)	0.049 (3)	−0.008 (3)	−0.011 (2)	−0.007 (3)
C22	0.058 (3)	0.077 (4)	0.051 (3)	−0.008 (3)	−0.003 (2)	−0.002 (3)
C23	0.060 (3)	0.045 (3)	0.057 (3)	−0.002 (2)	0.002 (2)	−0.009 (2)
C24	0.075 (4)	0.051 (3)	0.087 (4)	−0.004 (3)	0.014 (3)	−0.009 (3)
C25	0.096 (5)	0.056 (4)	0.125 (6)	−0.016 (3)	0.028 (4)	−0.008 (4)
C26	0.108 (5)	0.054 (4)	0.143 (7)	−0.012 (4)	0.016 (5)	−0.017 (4)
C27	0.154 (7)	0.066 (5)	0.126 (6)	−0.003 (5)	0.041 (6)	−0.044 (4)
C28	0.119 (5)	0.067 (4)	0.094 (4)	−0.008 (4)	0.042 (4)	−0.026 (4)
N1	0.052 (2)	0.060 (3)	0.052 (2)	0.001 (2)	−0.0102 (17)	−0.0085 (19)
N3	0.066 (5)	0.058 (6)	0.054 (5)	0.002 (4)	0.007 (4)	−0.012 (4)
O1	0.110 (3)	0.079 (3)	0.074 (3)	−0.031 (2)	0.014 (2)	−0.023 (2)
O2	0.080 (3)	0.110 (3)	0.055 (2)	−0.010 (2)	0.0100 (18)	0.006 (2)
O3	0.270 (15)	0.073 (6)	0.058 (5)	−0.038 (8)	−0.046 (7)	0.013 (4)
O4	0.095 (7)	0.110 (8)	0.193 (12)	−0.006 (6)	0.054 (7)	−0.038 (8)
P1	0.0522 (7)	0.0475 (7)	0.0416 (6)	−0.0017 (5)	−0.0034 (5)	−0.0020 (5)
S1	0.0551 (7)	0.0641 (9)	0.0713 (8)	0.0058 (6)	−0.0250 (6)	−0.0177 (7)
S2	0.0568 (7)	0.0928 (11)	0.0523 (7)	−0.0070 (7)	−0.0166 (6)	−0.0055 (7)
S3	0.0668 (8)	0.0698 (9)	0.0521 (7)	−0.0048 (7)	−0.0010 (6)	−0.0083 (6)
S5	0.0665 (19)	0.060 (2)	0.053 (2)	−0.0027 (18)	0.0020 (15)	0.0058 (16)
S4	0.0832 (19)	0.0563 (17)	0.0500 (15)	−0.0006 (14)	0.0157 (14)	−0.0023 (13)
S6	0.112 (2)	0.0629 (14)	0.0878 (16)	0	−0.0187 (13)	0
Sn1	0.0555 (3)	0.0525 (4)	0.0834 (4)	0	−0.0268 (3)	0

### Geometric parameters (Å, °)

**Table:**

C1—N1	1.290 (6)	C17—P1	1.785 (4)
C1—S1	1.748 (5)	C18—C19	1.381 (7)
C1—S2	1.751 (4)	C18—H18	0.93
C2—S3	1.746 (6)	C19—C20	1.385 (8)
C2—H2A	0.96	C19—H19	0.93
C2—H2B	0.96	C20—C21	1.363 (8)
C2—H2C	0.96	C20—H20	0.93
C3—S5	0.822 (12)	C21—C22	1.385 (7)
C3—N3	1.302 (15)	C21—H21	0.93
C3—S5^i^	1.750 (12)	C22—H22	0.93
C3—S4	1.763 (14)	C23—C28	1.364 (7)
C4—S6	1.621 (11)	C23—C24	1.378 (7)
C4—O4^i^	1.813 (16)	C23—P1	1.795 (5)
C4—O3	1.826 (16)	C24—C25	1.389 (8)
C4—H4A	1.0919	C24—H24	0.93
C4—H4B	0.9393	C25—C26	1.352 (9)
C4—H4C	0.9675	C25—H25	0.93
C5—C6	1.383 (7)	C26—C27	1.343 (10)
C5—C10	1.396 (7)	C26—H26	0.93
C5—P1	1.794 (5)	C27—C28	1.391 (9)
C6—C7	1.387 (7)	C27—H27	0.93
C6—H6	0.93	C28—H28	0.93
C7—C8	1.373 (9)	N1—S3	1.630 (4)
C7—H7	0.93	N3—S6	1.508 (8)
C8—C9	1.370 (9)	N3—S5	1.977 (9)
C8—H8	0.93	O1—S3	1.433 (4)
C9—C10	1.378 (7)	O2—S3	1.436 (4)
C9—H9	0.93	O3—S6	1.469 (8)
C10—H10	0.93	O4—S6	1.477 (9)
C11—C12	1.383 (6)	S1—Sn1	2.5125 (12)
C11—C16	1.394 (6)	S2—Sn1	2.5262 (15)
C11—P1	1.802 (5)	S5—C3^i^	1.750 (12)
C12—C13	1.384 (7)	S5—Sn1	2.441 (4)
C12—H12	0.93	S4—Sn1	2.646 (3)
C13—C14	1.361 (7)	S6—O3^i^	1.469 (8)
C13—H13	0.93	S6—O4^i^	1.477 (9)
C14—C15	1.369 (7)	S6—N3^i^	1.508 (8)
C14—H14	0.93	S6—C4^i^	1.621 (11)
C15—C16	1.367 (7)	Sn1—S5^i^	2.441 (4)
C15—H15	0.93	Sn1—S1^i^	2.5125 (12)
C16—H16	0.93	Sn1—S2^i^	2.5262 (15)
C17—C18	1.382 (7)	Sn1—S4^i^	2.646 (3)
C17—C22	1.388 (7)		
			
N1—C1—S1	117.7 (3)	C26—C25—C24	120.0 (6)
N1—C1—S2	127.2 (4)	C26—C25—H25	120
S1—C1—S2	115.1 (3)	C24—C25—H25	120
S3—C2—H2A	109.5	C27—C26—C25	120.5 (6)
S3—C2—H2B	109.5	C27—C26—H26	119.7
H2A—C2—H2B	109.5	C25—C26—H26	119.7
S3—C2—H2C	109.5	C26—C27—C28	120.7 (6)
H2A—C2—H2C	109.5	C26—C27—H27	119.7
H2B—C2—H2C	109.5	C28—C27—H27	119.7
S5—C3—N3	136.0 (15)	C23—C28—C27	119.5 (6)
S5—C3—S5^i^	94.5 (11)	C23—C28—H28	120.2
N3—C3—S5^i^	129.3 (10)	C27—C28—H28	120.2
N3—C3—S4	115.7 (9)	C1—N1—S3	122.0 (3)
S5^i^—C3—S4	115.0 (7)	C3—N3—O3	142.3 (10)
S6—C4—H4A	100.5	C3—N3—S6	126.4 (9)
O4^i^—C4—H4A	118.3	O3—N3—S5	140.2 (7)
O3—C4—H4A	126.2	S6—N3—S5	143.2 (6)
S6—C4—H4B	115	N3—O3—O4	94.0 (7)
C4^i^—C4—H4B	134.1	N3—O3—C4	102.9 (8)
O3—C4—H4B	115.4	C17—P1—C5	110.1 (2)
H4A—C4—H4B	118.2	C17—P1—C23	108.4 (2)
S6—C4—H4C	107	C5—P1—C23	109.6 (2)
C4^i^—C4—H4C	115.6	C17—P1—C11	106.7 (2)
O4^i^—C4—H4C	132.7	C5—P1—C11	112.4 (2)
H4A—C4—H4C	105.7	C23—P1—C11	109.6 (2)
H4B—C4—H4C	109.4	C1—S1—Sn1	86.82 (15)
C6—C5—C10	119.0 (4)	C1—S2—Sn1	86.33 (17)
C6—C5—P1	120.7 (4)	O1—S3—O2	116.2 (2)
C10—C5—P1	120.3 (4)	O1—S3—N1	111.5 (2)
C5—C6—C7	120.2 (5)	O2—S3—N1	111.1 (2)
C5—C6—H6	119.9	O1—S3—C2	108.7 (3)
C7—C6—H6	119.9	O2—S3—C2	108.5 (3)
C8—C7—C6	119.8 (6)	N1—S3—C2	99.5 (3)
C8—C7—H7	120.1	C3—S5—S4	142.7 (11)
C6—C7—H7	120.1	S4—S5—C3^i^	175.2 (4)
C9—C8—C7	120.8 (5)	S4—S5—N3	116.1 (3)
C9—C8—H8	119.6	C3^i^—S5—N3	64.0 (4)
C7—C8—H8	119.6	C3—S5—Sn1	127.1 (10)
C8—C9—C10	119.8 (5)	S4—S5—Sn1	89.6 (3)
C8—C9—H9	120.1	C3^i^—S5—Sn1	90.2 (5)
C10—C9—H9	120.1	N3—S5—Sn1	154.2 (3)
C9—C10—C5	120.4 (5)	S5—S4—Sn1	67.3 (3)
C9—C10—H10	119.8	C3—S4—Sn1	83.5 (4)
C5—C10—H10	119.8	O3^i^—S6—O3	175.2 (8)
C12—C11—C16	118.7 (4)	O3^i^—S6—O4	104.9 (7)
C12—C11—P1	122.6 (3)	O3—S6—O4	75.4 (7)
C16—C11—P1	118.6 (3)	O4^i^—S6—O4	174.6 (9)
C11—C12—C13	120.2 (4)	O3—S6—N3^i^	116.8 (5)
C11—C12—H12	119.9	O4^i^—S6—N3^i^	106.9 (5)
C13—C12—H12	119.9	O4—S6—N3^i^	77.4 (6)
C14—C13—C12	120.1 (5)	O3^i^—S6—N3	116.8 (5)
C14—C13—H13	119.9	O3—S6—N3	59.0 (5)
C12—C13—H13	119.9	O4^i^—S6—N3	77.4 (6)
C13—C14—C15	120.3 (5)	O4—S6—N3	106.9 (5)
C13—C14—H14	119.9	N3^i^—S6—N3	76.9 (7)
C15—C14—H14	119.9	O3^i^—S6—C4^i^	72.3 (6)
C16—C15—C14	120.5 (5)	O3—S6—C4^i^	112.1 (7)
C16—C15—H15	119.7	O4^i^—S6—C4^i^	103.7 (7)
C14—C15—H15	119.7	O4—S6—C4^i^	71.5 (6)
C15—C16—C11	120.2 (5)	N3^i^—S6—C4^i^	111.3 (6)
C15—C16—H16	119.9	N3—S6—C4^i^	170.6 (6)
C11—C16—H16	119.9	O3^i^—S6—C4	112.1 (7)
C18—C17—C22	119.3 (4)	O3—S6—C4	72.3 (6)
C18—C17—P1	119.5 (3)	O4^i^—S6—C4	71.5 (6)
C22—C17—P1	121.1 (4)	O4—S6—C4	103.7 (7)
C19—C18—C17	120.9 (5)	N3—S6—C4	111.3 (6)
C19—C18—H18	119.5	S5^i^—Sn1—S5	48.15 (19)
C17—C18—H18	119.5	S5^i^—Sn1—S1	97.70 (9)
C18—C19—C20	119.0 (6)	S5—Sn1—S1	100.94 (9)
C18—C19—H19	120.5	S5—Sn1—S1^i^	97.70 (9)
C20—C19—H19	120.5	S5^i^—Sn1—S2^i^	108.13 (10)
C21—C20—C19	120.5 (5)	S5—Sn1—S2^i^	152.94 (10)
C21—C20—H20	119.7	S1—Sn1—S2^i^	94.61 (5)
C19—C20—H20	119.7	S1^i^—Sn1—S2^i^	71.71 (4)
C20—C21—C22	120.7 (5)	S5^i^—Sn1—S2	152.94 (10)
C20—C21—H21	119.7	S5—Sn1—S2	108.13 (10)
C22—C21—H21	119.7	S1—Sn1—S2	71.71 (4)
C21—C22—C17	119.5 (5)	S1^i^—Sn1—S2	94.61 (5)
C21—C22—H22	120.2	S5—Sn1—S4^i^	71.14 (14)
C17—C22—H22	120.2	S1—Sn1—S4^i^	96.17 (7)
C28—C23—C24	119.5 (5)	S2^i^—Sn1—S4^i^	85.36 (8)
C28—C23—P1	119.3 (4)	S2—Sn1—S4^i^	167.66 (7)
C24—C23—P1	121.0 (4)	S1—Sn1—S4	97.69 (8)
C23—C24—C25	119.8 (5)	S1^i^—Sn1—S4	96.17 (7)
C23—C24—H24	120.1	S2^i^—Sn1—S4	167.66 (7)
C25—C24—H24	120.1	S2—Sn1—S4	85.36 (8)
			
C10—C5—C6—C7	−1.3 (8)	C3^i^—C3—N3—S6	5.2 (18)
P1—C5—C6—C7	−179.2 (4)	S5^i^—C3—N3—S6	5.2 (15)
C5—C6—C7—C8	0.6 (9)	S4—C3—N3—S6	−175.4 (6)
C6—C7—C8—C9	0.3 (9)	S4—C3—N3—N3^i^	−177.0 (8)
C7—C8—C9—C10	−0.5 (9)	C3^i^—C3—N3—S5	−173 (3)
C8—C9—C10—C5	−0.2 (8)	S5^i^—C3—N3—S5	−173 (3)
C6—C5—C10—C9	1.1 (7)	S4—C3—N3—S5	6.0 (12)
P1—C5—C10—C9	179.1 (4)	C18—C17—P1—C5	−70.6 (4)
C16—C11—C12—C13	−1.2 (7)	C22—C17—P1—C5	112.8 (4)
P1—C11—C12—C13	−176.7 (4)	C18—C17—P1—C23	169.5 (4)
C11—C12—C13—C14	0.5 (8)	C22—C17—P1—C23	−7.1 (4)
C12—C13—C14—C15	0.2 (8)	C18—C17—P1—C11	51.6 (4)
C13—C14—C15—C16	−0.3 (9)	C22—C17—P1—C11	−125.0 (4)
C14—C15—C16—C11	−0.4 (9)	C6—C5—P1—C17	2.9 (5)
C12—C11—C16—C15	1.1 (8)	C10—C5—P1—C17	−175.1 (4)
P1—C11—C16—C15	176.8 (4)	C6—C5—P1—C23	122.0 (4)
C22—C17—C18—C19	0.3 (7)	C10—C5—P1—C23	−55.9 (4)
P1—C17—C18—C19	−176.3 (4)	C6—C5—P1—C11	−115.9 (4)
C17—C18—C19—C20	0.0 (8)	C10—C5—P1—C11	66.2 (4)
C18—C19—C20—C21	−0.1 (8)	C28—C23—P1—C17	−71.1 (5)
C19—C20—C21—C22	−0.2 (8)	C24—C23—P1—C17	103.7 (5)
C20—C21—C22—C17	0.5 (8)	C28—C23—P1—C5	168.7 (5)
C18—C17—C22—C21	−0.5 (7)	C24—C23—P1—C5	−16.5 (5)
P1—C17—C22—C21	176.1 (4)	C28—C23—P1—C11	45.0 (5)
C28—C23—C24—C25	0.9 (9)	C24—C23—P1—C11	−140.3 (4)
P1—C23—C24—C25	−173.9 (5)	C12—C11—P1—C17	−157.2 (4)
C23—C24—C25—C26	0.7 (11)	C16—C11—P1—C17	27.3 (5)
C24—C25—C26—C27	−1.0 (13)	C12—C11—P1—C5	−36.4 (5)
C25—C26—C27—C28	−0.3 (14)	C16—C11—P1—C5	148.1 (4)
C24—C23—C28—C27	−2.2 (10)	C12—C11—P1—C23	85.7 (5)
P1—C23—C28—C27	172.7 (6)	C16—C11—P1—C23	−89.8 (4)
C26—C27—C28—C23	2.0 (13)	N1—C1—S1—Sn1	178.7 (4)
S1—C1—N1—S3	179.5 (2)	S2—C1—S1—Sn1	−2.4 (2)
S2—C1—N1—S3	0.7 (6)	N1—C1—S2—Sn1	−178.8 (4)
S5—C3—N3—O3	94 (2)	S1—C1—S2—Sn1	2.4 (2)
C3^i^—C3—N3—O3	−80 (2)	C1—N1—S3—O1	−60.3 (5)
S4—C3—N3—O3	99.6 (15)	C1—N1—S3—O2	71.0 (5)
S5—C3—N3—S6	178.6 (15)	C1—N1—S3—C2	−174.8 (5)

Symmetry codes: (i) −*x*, *y*, −*z*+1/2.

### Hydrogen-bond geometry (Å, °)

**Table:**

*D*—H···*A*	*D*—H	H···*A*	*D*···*A*	*D*—H···*A*
C2—H2B···O4^ii^	0.96	2.35	3.284 (13)	166
C16—H16···O3^iii^	0.93	2.60	3.2203 (10)	125
C19—H19···O1^iv^	0.93	2.47	3.296 (7)	148
C28—H28···S4^iii^	0.93	2.69	3.345 (5)	128

Symmetry codes: (ii) *x*+1/2, *y*−1/2, *z*; (iii) *x*, −*y*+1, *z*+1/2; (iv) *x*, −*y*, *z*+1/2.
